# Giant subcutaneous lymphangioma of the abdominal wall in childhood

**DOI:** 10.11604/pamj.2021.39.104.30068

**Published:** 2021-06-03

**Authors:** Rohan Kumar Singh, Gaurav Vedprakash Mishra

**Affiliations:** 1 Department of Radiodiagnosis, Jawaharlal Nehru Medical College, Datta Meghe Institute of Medical Sciences, Sawangi (Meghe), Wardha, India

**Keywords:** Cystic lymphangioma, subcutaneous, ultrasonography

## Image in medicine

A 9-year-old male child was brought by his father to the emergency department of the hospital with chief complaints of swelling on the left half of the abdomen since birth. The father of the child told that he had swelling of small size at birth which started gradually increasing in size for 18 months and then it was static up to 6 years of age. Later it again started increasing in size and now it has occupied the entire left abdomen. On clinical examination, the swelling was soft in consistency, non-tender with mild reddish-blue discolouration of overlying skin and no local rise of temperature. There was no pus discharge and itching over the swelling. For further investigation child was referred to the department of radiodiagnosis for contrast-enhanced computed tomography and ultrasonography of the abdomen. On computed tomography multiple varied size hypodense areas, most of them communicating with each other seen in the subcutaneous plane of anterior abdominal wall more on left side extending from left hypochondrium to left lumbar region inferiorly and crossing midline reaching right hypochondrium laterally on the posterolateral aspect of the abdominal wall. Few areas showed hyperdense content within with no significant enhancement on all post-contrast phases. On ultrasound, multiple cystic anechoic areas with internal debris independent part and showing no vascularity were noted.

**Figure 1:**
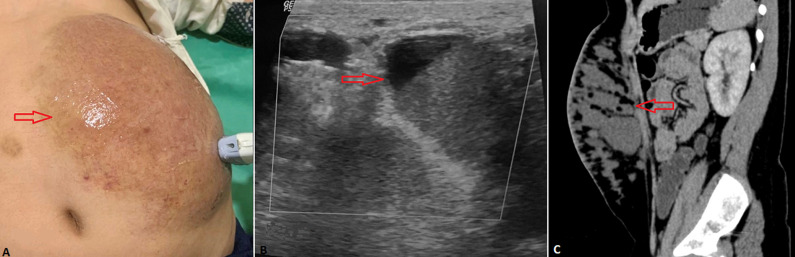
A) large mild reddish-blue smooth lesion occupying the left side of the abdomen (red arrow); B) ultrasound image of the lesion showing multiple cystic lesions with internal debris within (red arrow); C) CT scan sagittal section shows multiple soft tissue density lesion communicating with each other in the subcutaneous plane of abdomen (red arrow); findings consistent with abdominal lymphatic malformation- lymphangioma

